# Normalization Benefits Microarray-Based Classification

**DOI:** 10.1155/BSB/2006/43056

**Published:** 2006-08-24

**Authors:** Jianping Hua, Yoganand Balagurunathan, Yidong Chen, James Lowey, Michael L Bittner, Zixiang Xiong, Edward Suh, Edward R Dougherty

**Affiliations:** 1 Computational Biology Division, Translational Genomics Research Institute, Phoenix, AZ 85004, USA; 2 Genetics Branch, Center for Cancer Research, National Cancer Institute, National Institutes of Health, Bethesda, MD 20892-2152, USA; 3 Department of Electrical & Computer Engineering, Texas A&M University, College Station, TX 77843, USA

## Abstract

When using cDNA microarrays, normalization to correct labeling bias is a common preliminary step before further data analysis is applied, its objective being to reduce the variation between arrays. To date, assessment of the effectiveness of normalization has mainly been confined to the ability to detect differentially expressed genes. Since a major use of microarrays is the expression-based phenotype classification, it is important to evaluate microarray normalization procedures relative to classification. Using a model-based approach, we model the systemic-error process to generate synthetic gene-expression values with known ground truth. These synthetic expression values are subjected to typical normalization methods and passed through a set of classification rules, the objective being to carry out a systematic study of the effect of normalization on classification. Three normalization methods are considered: offset, linear regression, and Lowess regression. Seven classification rules are considered: 3-nearest neighbor, linear support vector machine, linear discriminant analysis, regular histogram, Gaussian kernel, perceptron, and multiple perceptron with majority voting. The results of the first three are presented in the paper, with the full results being given on a complementary website. The conclusion from the different experiment models considered in the study is that normalization can have a significant benefit for classification under difficult experimental conditions, with linear and Lowess regression slightly outperforming the offset method.

## 

[1,2,3,4,5,6,7,8,9,10,11,12,13,14,15,16,17,18,19,20]
